# Does head and cervical posture correlate to malocclusion? A systematic review and meta-analysis

**DOI:** 10.1371/journal.pone.0276156

**Published:** 2022-10-25

**Authors:** Houli Peng, Weihan Liu, Lanxin Yang, Wenjie Zhong, Yuanyuan Yin, Xiang Gao, Jinlin Song

**Affiliations:** 1 College of Stomatology, Chongqing Medical University, Chongqing, China; 2 Chongqing Key Laboratory of Oral Diseases and Biomedical Sciences, Chongqing, China; 3 Chongqing Municipal Key Laboratory of Oral Biomedical Engineering of Higher Education, Chongqing, China; 4 Department of orthodontics, Chongqing University Three Gorges Hospital, Chongqing, China; University of Catanzaro, ITALY

## Abstract

**Background:**

The association of head and cervical posture with malocclusion has been studied for many years. Despite extensively encouraging researches, no conclusive evidence has been reached for clinical application.

**Objective:**

To identify the question “Does head and cervical posture correlate to malocclusion?”, a systematic review and meta-analysis based on the available studies were carried out (PROSPERO registration number: CRD42022319742).

**Methods:**

A search of PubMed, Embase, Cochrane Library, and the grey literature was performed without language restrictions. The study screening, data extraction, risk-of-bias evaluation and methodological quality assessment were performed by two independent investigators. When a disagreement arose, a third author was consulted.

**Results:**

6 original cross-sectional studies involving 505 participants were included, which were of moderate methodological quality. NL/VER in Class Ⅱ group and NL/CVT in Class Ⅲ group showed significant differences compared to Class Ⅰ group, but no significant differences were observed in most of the variables like NSL/VER, OPT/CVT, OPT/HOR, CVT/HOR, NSL/OPT, NSL/CVT, NL/OPT in Class Ⅱ and Ⅲ groups.

**Conclusions:**

The results suggested that the current research evidence is not sound enough to prove the association of head and cervical posture with sagittal malocclusion. Better controlled design and a larger sample size are required for clarifying this question in future study.

## Introduction

Poor posture of head and neck is considered to be one of the major causes for myofunctional disorders in craniofacial region. When abnormal posture of head and neck takes place during active growth stage of patient, the normal development of craniofacial structure may be disrupted due to biomechanical and anatomical connection between neck muscles and craniofacial structure [1–4]. Moreover, according to the previous epidemiologic studies, patients suffering from neck disorders showed a higher percentage of craniomandibular disorders, which are often accompanied by malocclusion [5–8]. Therefore, a hypothesis that head and cervical posture correlates to malocclusion was put forward and widely reported in the field of orthodontics and orthopedics [9]. For example, emerging evidence reported that children with Class Ⅱ malocclusion, which were characterized by a convex profile with a retrognathic mandible and/or a prognathic maxilla, have an obviously higher head extension upon the spinal column, while a significant lower cervical lordosis angle was observed in subjects with Class Ⅲ malocclusion, which showed a concave profile with a prognathic mandible and/or a retruded maxilla, suggesting that an alteration in cranio-cervical posture has a close association with sagittal malocclusion [10–17]. Therefore, various conservative and physical therapies for abnormal head and cervical posture correction, such as postural continuous monitoring, transcutaneous electrical nerve stimulation (TENS), bracing, and smart posture corrective orthosis, show a great potential for prevention of the development of sagittal malocclusion [18–20].

Despite encouraging results obtained from a number of studies, the hypothesis that the correlation of head and cervical posture with malocclusion was still challenged by some researchers [9, 21–23]. A typical viewpoint is that most of current studies neglected the possible confounding effect of age [24, 25], which may contribute to the change in degree of cervical lordosis, so the abnormal jaw relation may be ascribed to the developmental disorder rather than poor cervical posture. Considering these debates, the evidence for a clear correlation of cranio-cervical posture with malocclusion is in great demand, because proper understanding their relationship is of fundamental importance for diagnosis, prevention and treatment of malocclusion in patients with abnormal cranio-cervical posture [26]. To our knowledge, a review about the relationship postural disorders associated with dentofacial morphology was published by Huggare in 1998 [27]. A series of systematic reviews about the relationship of occlusion with posture as well as the relationship of cervical posture associated with craniofacial morphology were reported during 1999–2017 [1, 23, 26, 28, 29]. However, these reviews were mainly based on discussion of previous anecdotes, case reports and epidemiological studies, lacking quantitative evaluation on relevant topic. Different from previous reviews, the purpose of the present study is to focus on elucidating the relationship of head and cervical posture with malocclusion, which is of particular interest to the practitioners of orthodontics and orthopedics. Moreover, qualitative and quantitative assessments are simultaneously performed on the currently existing evidence to improve the validity and reliability of conclusion.

## Materials and methods

### Protocol and registration

The protocol of this meta-analysis was registered in the US National Institute of Health’s (NIH; Bethesda, Maryland, USA) International Prospective Register of Systematic Reviews research database (https://www.crd.york.ac.uk/PROSPERO, Protocol: CRD42022319742.) and was reported in accordance with the Preferred Reporting Items for Systematic Reviews and Meta-Analyses (PRISMA) statement (see **Table A** in the **S1 Appendix**).

### Search strategy

Electronic databases including Embase, Pubmed and Cochrane library, and the gray literature by Google Scholar search were searched up to April 2022 without language limitation. The search strategy consisted of Medical Subject Headings (MeSH), a mix of free-text terms and keywords. Relevant free terms and keywords applied for malocclusion are “Angle’s classification”, “angle class ii malocclusion”, “angle class iii malocclusion”, “jaw occlusion” *etc*. The terms used for head and cervical posture included “head posture”, “head position”, “cervical posture”, “cervical position”, “neck posture”, “craniocervical posture”, “cervical curvature”, “cervical lordosis”, “cervical vertebrae”. Moreover, a manual search was also performed on the relevant references. The search strings were built (see **Table B** in the **S1 Appendix**).

After searching the above databases, all identified documents were imported into Endnote software. Two authors independently identified the relevant studies according to the eligibility criteria. Duplicates and irrelevant articles which did not meet the inclusion criteria were removed. When discrepancies occurred, a third author resolved the disagreement in accordance with the inclusion criteria until a final consensus was achieved.

### Eligibility criteria

All studies were evaluated for eligibility based on the following PICO model: (P) the patients diagnosed with malocclusion without a history of orthodontic treatment were included; (I) not applicable; (C) the group of Class Ⅰ malocclusion (subjects with a normal sagittal jaw relationship) was applied as the control to assess the relationship of cranio-cervical posture with Class Ⅱ and Ⅲ malocclusion; (O) Outcome measures consisted of craniovertical angles, cervicohorizontal angles, craniocervical angles and cervical curvature obtained by cephalometric analysis. In addition, all postural variables were measured in natural head position (NHP) to reduce the influence of intracranial reference planes like Sella Nasion (SN) and Frankfort Horizontal (FH) planes, which was adopted worldwide because of its good stability and reproducibility [30–33]. Observational studies including cross-sectional studies, cohort studies, and case-control studies were screened out for evaluation. Other factors such as gender, age and ethnicity were not restricted. Books, case reports, reviews, animal studies were excluded. The detailed eligibility criteria were listed as **Table 1**.

**Table 1:** Eligibility criteria.

Inclusion criteria	Exclusion criteria
1. Patients diagnosed with malocclusion 2. Complete permanent dentition (third molar is not taken into account) 3. Observational studies such as cross-sectional studies, cohort studies, and case-control studies evaluating the correlation of head and cervical posture with malocclusion 4. Natural head position 5. Outcome measures including craniovertical angle, cervicohorizontal angle, craniocervical angle and cervical curvature 6. No history of surgery, orthodontic treatment or physiotherapy	1. Poor cervical posture due to specific underlying pathologies such as congenital disorders of the craniofacial complex, disease of muscles, and temporomandibular joint disorders, cleft lip, and palate 2. Animal experimental studies 3. Data sources unclear, review, case reports and meta-analysis literature, repeat publication (select the first to publish)

### Data items and collection

Two researchers independently performed the data extraction according to the eligibility criteria. When disagreement arose, two researchers discussed until consensus was achieved. To ensure that included studies are reliable for the critical appraisal, the published paper should provide enough information to meet the criterion, which comprised race, sample size, age, gender, assessment method, and relevant outcome indicators.

In the present study, the reference points and lines were traced according to the analytical method described by Solow and Tallgren [34]. Nine cephalometric variables representing the craniovertical, cervicohorizontal and craniocervical postural relationships and the cervical column curvature (NSL/VER, NL/VER, OPT/HOR, CVT/HOR, NSL/OPT, NSL/CVT, NL/OPT, NL/CVT, OPT/CVT) were collected and analyzed (see **Fig A** in the **S1 Appendix**). The head posture was defined as the craniovertical angle between the head and the true vertical (NSL/VER and NL/VER). The cervical column inclination was determined by cervicohorizontal angles, which formed by the upper cervical and the true horizontal (OPT/HOR and CVT/HOR). The craniocervical posture was determined by craniocervical angles, which formed by the posture of head and cervical column (NSL/OPT, NSL/CVT, NL/OPT, NL/CVT). The angle formed by OPT and CVT indicates cervical curvature. The descriptions of reference points, lines, and angles used in our study were summarized (see **Table C** in the **S1 Appendix**).

### Risk of bias and quality assessment

Two authors independently evaluated the quality of selected articles based on an adapted form of Joanna Briggs Institute (JBI) critical appraisal checklist [35] and compared the results of evaluation until agreement was reached. Discrepancies in the assessment were resolved by discussion until consensus was reached. Quality assessment was focused on the purpose of study, sample selection, inclusion and exclusion criteria, characteristics of subject, reliability and validity of outcome measurement, appropriate external validity, ethical issue, statistical method, and outcome and research value.

According to JBI critical appraisal tool, each topic would be scored individually. A score of 0 indicates no mention of the topic, 1 point signifies a brief mention of the clause but no specificity, and 2 points indicate not only a reference to the clause but also a detailed description of it. Eventually, a score higher than 70% of the total score is typically defined as a high-quality study. Considering that there is no standard for the range of medium and low-quality scores, two reviewers defined the quality score range based on the calculation method. Studies scoring 14–20 are considered to be of high quality, 8–14 of moderate quality, and less than 8 of low quality.

### Data synthesis

The collected data were meta-analyzed using Review Manager Software (RevMan, version 5.4; Nordic Cochrane Center, Cochrane Collaboration, Denmark). The continuous variable data were calculated by the mean difference (MD) and its 95% confidence interval (95% CI), and *p* < 0.05 was considered as statistically difference. The heterogeneity among studies was evaluated by *I*^2^ statistic, which describes true variation across studies as a percentage, with values of 25%, 50%, and 75% considered low, moderate, and high heterogeneity, respectively [36, 37]. If there was no significant heterogeneity (*p* ≥ 0.05, *I*^2^ ≤ 50%), the meta-analysis was expressed using a fixed-effects model. If there was significant heterogeneity (p < 0.05, *I*^2^ > 50%), the sources of heterogeneity were eliminated for further analysis. When heterogeneity still existed, a random-effects model was adopted. Publication bias was not tested due to small number of the included studies.

## Results

### Study selection and characteristics

**Fig 1:**
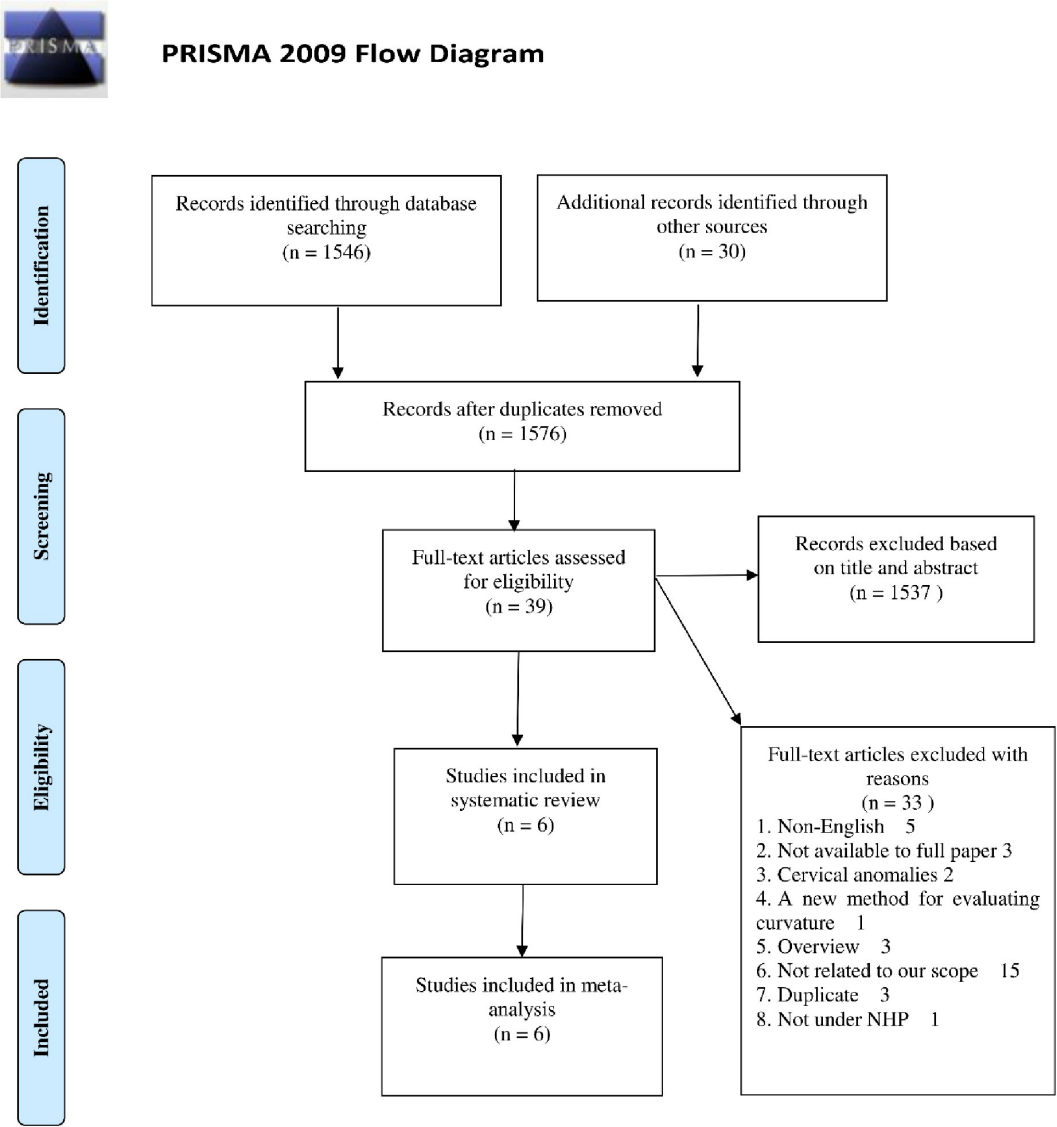
PRISMA flow chart for study selection.

The search of selected databases provided a total of 1460 publications. After removing duplicates and ineligible articles, 39 articles were selected as potential studies based on the evaluation of their abstracts. Finally, a total of 6 studies involving 505 individuals were included for review and meta-analysis after reading full-text [38–43]. No other observational studies like case-control or cohort studies that met the inclusion criteria were found. 3 of the 6 articles measured head posture (NSL/VER, NL/VER), 3 of the 6 studies measured cervical curvature (OPT/CVT), all of the 6 studies measured cervical spine inclination (OPT/HOR, CVT/HOR), 2 of the 6 studies measured craniocervical posture (NSL/OPT, NSL/CVT, NL/OPT, NL/CVT). The process of study selection was presented in **Fig 1**.

All included publications were cross-sectional studies. It was noted that in one of the included studies, the posture variables were discussed in two age groups (9–11 years old, and above 18 years old), respectively. In this study, the average age of all subjects was 13.49 years, and the sample size of group between 9 and 11 years old was larger than the other. Therefore, the former age group (9–11 years old) was selected for data collection and analysis [41].

In addition, there are some questions remaining to be answered including uneven sample size distribution, inappropriate study design, and possible confounders. For example, there were only 6 subjects in Class Ⅲ group, which had 37 fewer subjects than Class Ⅱ group [38]. Moreover, potential confounding factors such as ethnicity, age, gender were not sufficiently taken into account in the included studies. The participants came from four different countries. One study did not mention the nationality of subjects, and only two studies showed that subjects were from the same country, Iran [38, 39, 41]. Only one [41] study analyzed the data from different age subgroups, while the other five studies analyzed data without age distinction, thus leading to a greater heterogeneity. The main characteristics of the included articles were displayed in **Table 2**.

**Table 2:** Characteristics of included studies.

Region	participants	Age(years)	Gender	Assessment	Variables				Conclusions
					Cervical curvature	CVA	CHA	CCA	
Liu *et al*. (2016)									
China	90 participants Class Ⅰ: 30 Class Ⅱ: 30 Class Ⅲ: 30	11–14	Male: 45 Female: 45	Lateral cephalometric radiographs	OPT/CVT	NSL/VER NL/VER	OPT/HOR	NSL/OPT NSL/CVT NL/OPT NL/CVT	Tendencies: skeletal Class Ⅱ: more extended head; skeletal Class Ⅲ: flexed head
Bernal *et al*. (2017)									
-	107 participants Class Ⅰ: 58 Class Ⅱ: 43 Class Ⅲ: 6	6–11	Male: 52 Female: 55	Lateral cephalometric radiographs	-	NSL/VER NL/VER	OPT/HOR	-	The relationship between cervical postural variables and different malocclusion: no differences
Qidar *et al*. (2017)									
Indian-controlled Kashmir area	90 participants Class Ⅰ: 32 Class Ⅱ: 31 Class Ⅲ: 27	15–35	Male: 43 Female: 47	Lateral radiographs	OPT/CVT	-	OPT/HOR	-	1. Skeletal Class Ⅱ: OPT/CVT increased 2. Head was backwardly positioned in Class Ⅰ compared to Class Ⅲ
Tauheed *et al*. (2019)									
Pakistani	63 participants Class Ⅰ: 22 Class Ⅱ: 21 Class Ⅲ: 20	11–22	Male: 25 Female: 38	Lateral radiographs	OPT/CVT	-	OPT/HOR	-	1. Skeletal malocclusion differs in their cervical postures, especially cervical curvature. 2. Skeletal Class Ⅲ: straighter cervical columns
Hedayati *et al*. (2013)									
Iran	102 participants Class Ⅰ: 32 Class Ⅱ: 40 Class Ⅲ: 30	15–18	-	Lateral radiographs	-	NSL/VER NL/VER	OPT/HOR	NSL/OPT NSL/CVT NL/OPT NL/CVT	Tendencies: more forward head posture and incline their head in toward the chest (ventral) in skeletal Class Ⅲ
Nik *et al*. (2011)									
Iran	53 participants Class Ⅰ: 7 Class Ⅱ: 24 Class Ⅲ: 22	9–11	-	Lateral radiographs	OPT/CVT	-	OPT/HOR	-	1.Class Ⅱ: cervical column posture 2. Age have not affected the curvature and cervical posture

Abbreviations: craniovertical angles (CVA); cervicohorizontal angles (CHA), craniocevical angles (CCA).

### Risk of bias within studies

Results from the JBI quality assessment tool were shown in **Table 3**. Since none of included studies provided sufficient information to assess the quality and conformed to the principle of blinded randomized controlled trial, most of studies scored at 8–10 without reaching the required scores of high-quality standards.

**Table 3:** Quality assessment of included studies.

Studies	Proposition	sample selection	criteria/exclusions	Sample features	reliability and validity	Data collection	Ethics	statistical methods	results	research values	scores	Final rating
**Bernal, *et al*. (2017)**	2	0	1	1	1	1	1	1	1	1	10	Moderate
**Qadir, *et al*. (2017)**	2	0	1	1	0	0	0	1	1	1	8	Moderate
**Tauheed, *et al*. (2019)**	2	0	1	1	1	1	0	1	1	1	9	Moderate
**Hedayati, *et al*. (2013)**	1	0	1	1	1	1	0	1	1	1	8	Moderate
**Nik *et al*. (2011)**	1	0	1	1	1	1	0	1	1	1	8	Moderate
**Liu *et al*. (2016)**	2	0	1	1	1	1	1	1	1	1	10	Moderate

Scoring of quality assessment: High quality, 14–20; Moderate quality, 8–14; Low quality, <8

### Meta-analysis

**Fig 2:**
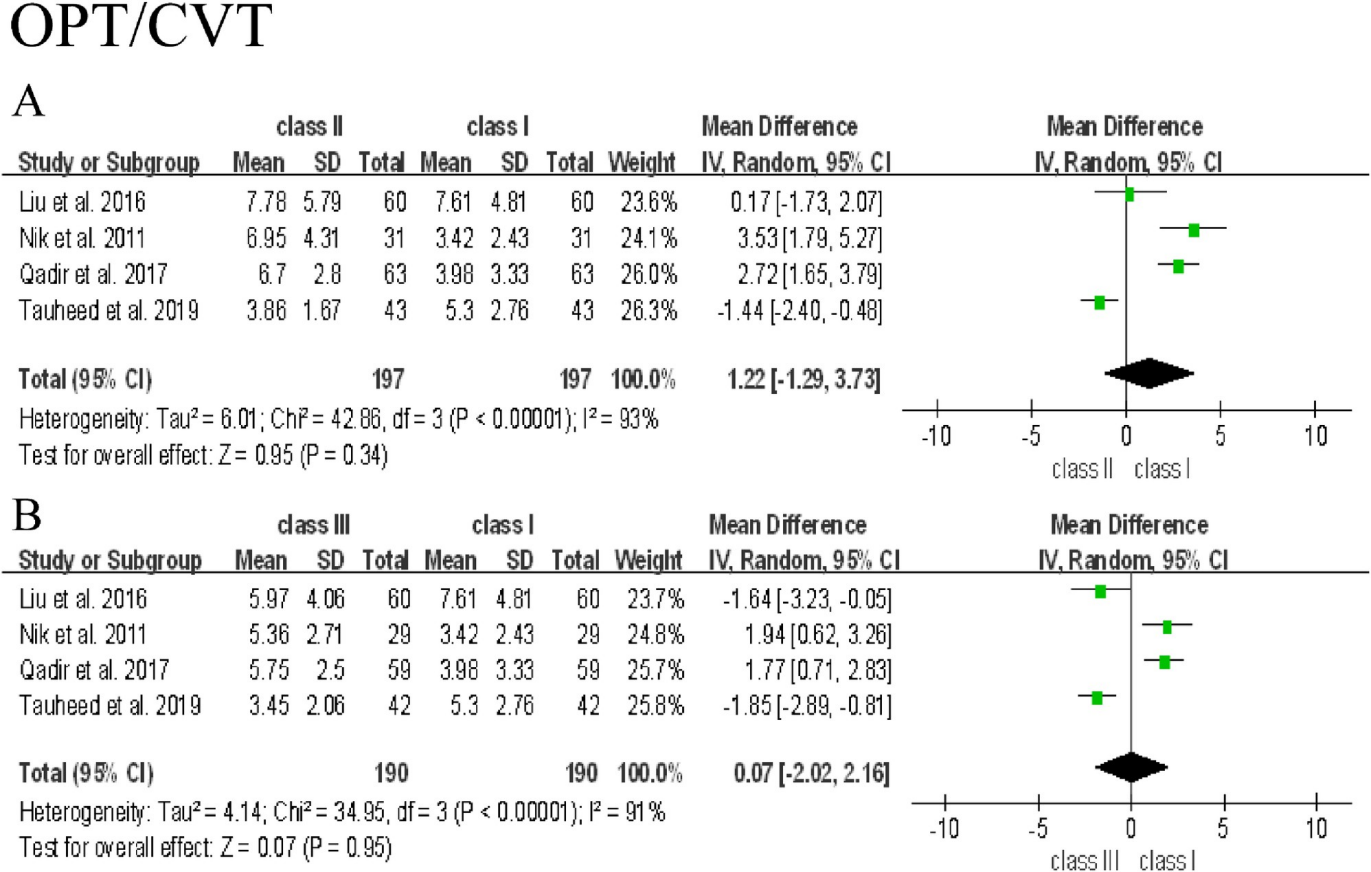
Forest plot of the association between cervical curvature and Class Ⅰ, Ⅱ, and Ⅲ malocclusion.

**Fig 3:**
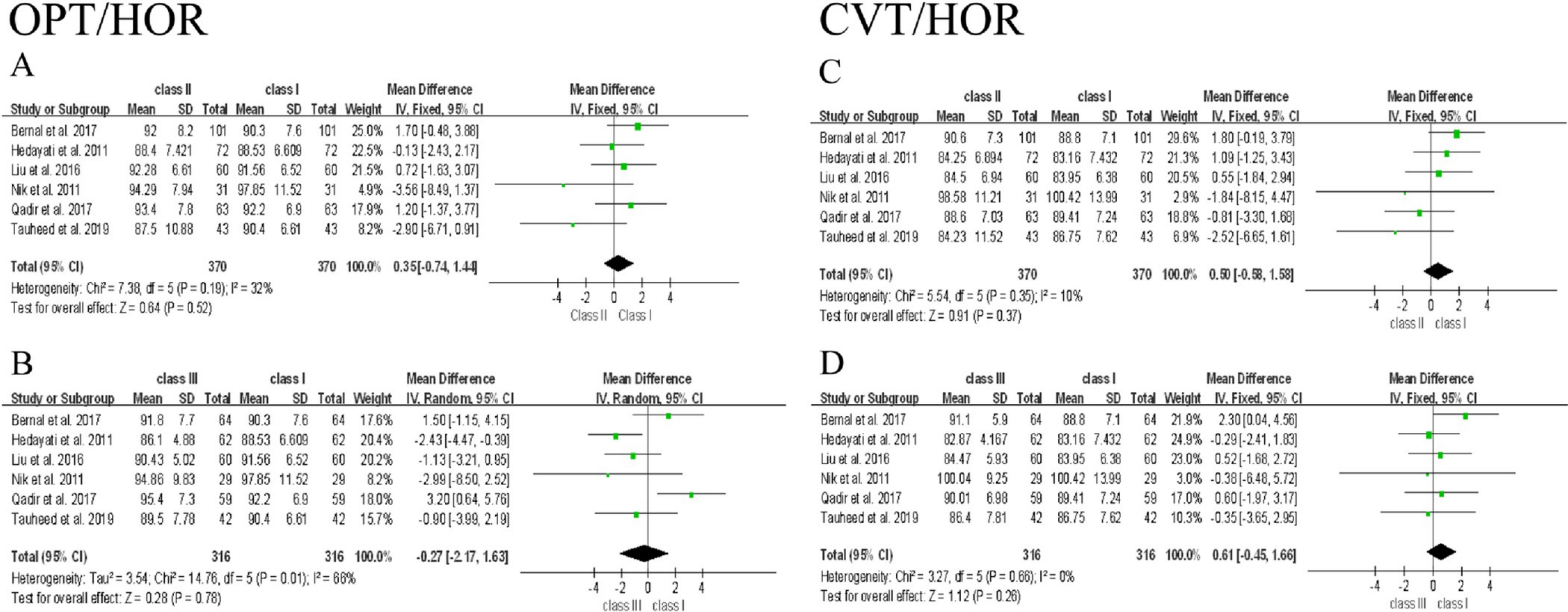
Forest plot of the association between cervical inclination and Class Ⅰ, Ⅱ, and Ⅲ malocclusion.

**Fig 4:**
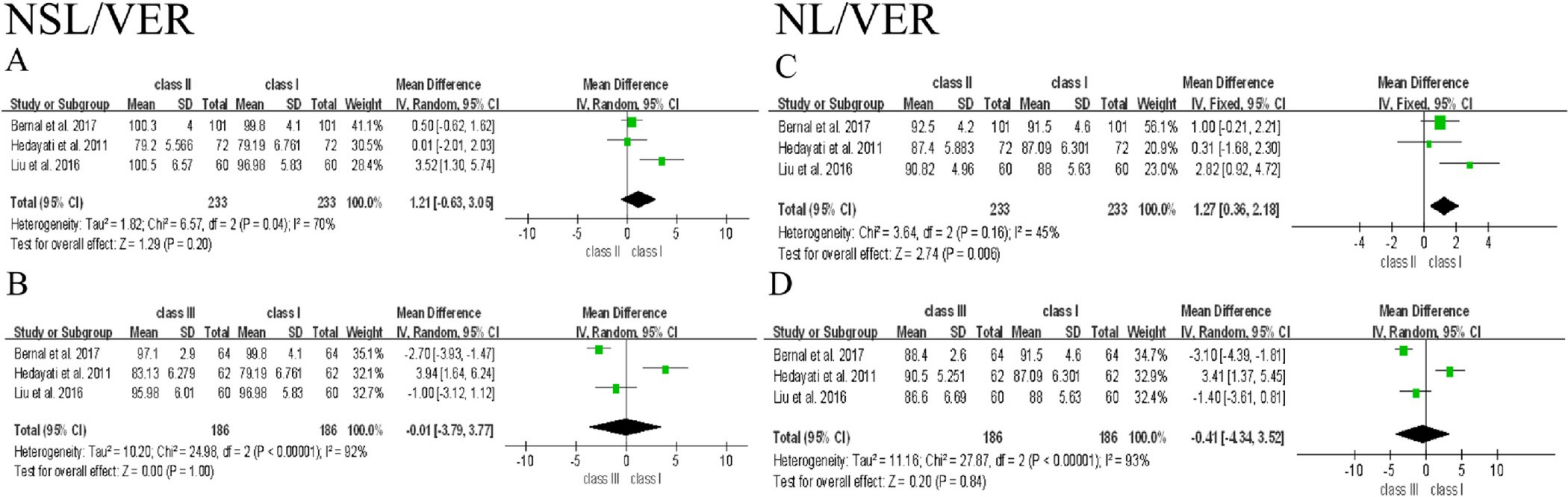
Forest plot of the association between head posture and Class Ⅰ, Ⅱ, and Ⅲ malocclusion.

**Fig 5:**
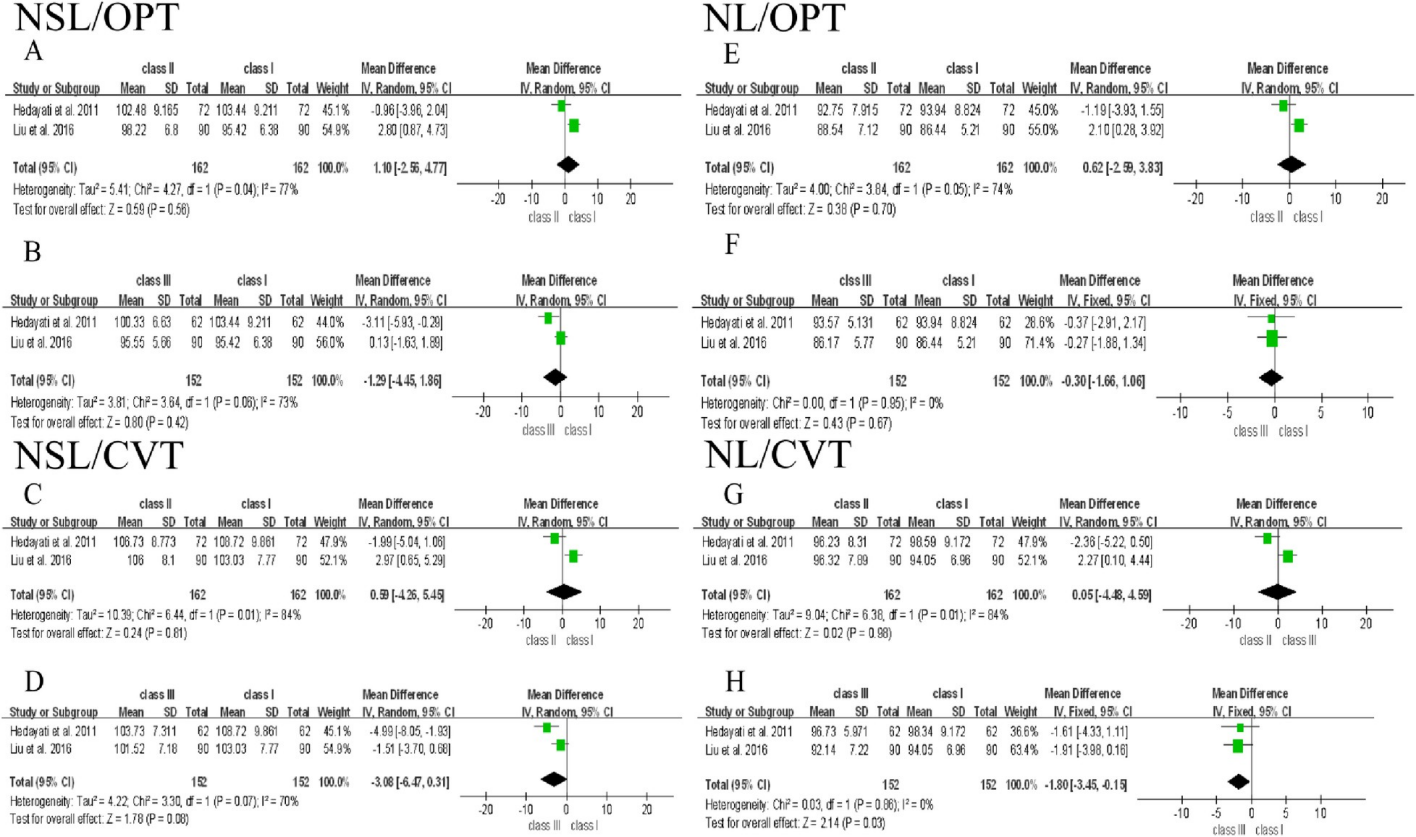
Forest plot of the association between craniocervical posture and Class Ⅰ, Ⅱ, and Ⅲ malocclusion.

The meta-analysis was conducted based on the data from 6 included studies. Since postural variables used in each study were different, they were discussed separately in this analysis. The difference of 9 posture variables among participants with Class Ⅰ, Ⅱ, and Ⅲ malocclusion and the forest plot of their association were shown as below, and Class Ⅰ group was used as control **(Figs 2–5)**. To clarify the difference of overall postural variables among patients with Class Ⅰ, Ⅱ, and Ⅲ malocclusion, the statistical heterogeneity between studies was explored and assessed using *I*^2^ test. Subgroup analysis was not performed, because most of studies did not distinguish the differences of confounding factors like age, gender and ethnicity among groups.

#### Cervical curvature

The value of OPT/CVT showed no significant differences in Class Ⅱ and Ⅲ groups compared to Class Ⅰ group due to the presence of a significant high heterogeneity (*I*^2^ = 93%, *p* = 0.34 in Class Ⅱ group; *I*^2^ = 91%, *p* = 0.95 in Class Ⅲ group) **(Fig 2)**. After removing the source of heterogeneity in Class Ⅱ group, the value of OPT/CVT in Class Ⅱ group showed a significant difference compared to Class Ⅰ group (*I*^2^ = 0%; MD = 2.94, 95% CI [2.03 to 3.86]; *p* < 0.00001), implying that the study reported by Tauheed, as the source of heterogeneity, has a negative impact on the result of OPT/CVT.

#### Cervical column inclination

All six articles measured cervical column inclination changes (OPT/HOR, CVT/HOR) in Class Ⅰ, Ⅱ, and Ⅲ malocclusion [38–43]. A strong heterogeneity was found in Class Ⅲ group of OPT/HOR **(Fig 3B)**, and then the random effect model was applied. When the source of heterogeneity was excluded [42], the value of OPT/HOR in Class Ⅲ group still showed no significant differences (When not excluded: *I*^2^ = 66%, MD = -0.27, 95% CI [-2.17 to 1.63]; *p* = 0.78. After excluding heterogeneity: *I*^2^ = 31%, MD = -1.04, 95% CI [-2.49 to 0.41]; *p* = 0.16) **(Fig 3B)**. Despite weak **(Fig 3A and 3C)**, even no heterogeneity **(Fig 3D)** among the groups, the result of OPT/HOR in Class Ⅱ group as well as the changes of CVT/HOR both in Class Ⅱ and Class Ⅲ groups showed no significant differences compared to Class Ⅰ group.

#### Head posture

Half of the studies measured the head posture (NSL/VER, NL/VER) in Class Ⅱ and Ⅲ groups **(Fig 4)** [38–40]. However, a strong heterogeneity was found between Class Ⅱ and Ⅲ groups **(Fig 4A, 4B and 4D)**. For NSL/VER variable, there was a strong heterogeneity in Class Ⅱ group (heterogeneity: *I*^2^ = 70%), so the random effect model was adopted. No matter the heterogeneous sources were excluded or not, the value of NSL/VER showed no significant differences compared to Class Ⅰ group [40], (When not excluded, *I*^2^ = 70%, MD = 1.21, 95% CI [-0.63 to 3.05]; p = 0.2. When excluded, *I*^2^ = 0%, MD = 0.39, 95% CI [-0.59 to 1.36]; p = 0.44) **(Fig 4A)**. Likewise, a strong heterogeneity was detected in Class Ⅲ group of NSL/VER (*I*^2^ = 92%). When the heterogeneous sources were excluded, the NSL/VER value showed a significant difference in Class Ⅲ group compared to Class Ⅰ group (When not excluded, MD = -0.01, 95% CI [-3.79 to 3.77]; *p* = 1.00. After excluding, *I*^2^ = 46%, MD = -2.27, 95% CI [-3.34 to -1.21]; *p* < 0.0001 **(Fig 4B)** [39].

For NL/VER variable, a significant difference and a moderate heterogeneity was showed Class Ⅱ group compared to Class Ⅰ group (*I*^2^ = 45%, MD = 1.27, 95% CI (0.36 to 2.18), *p* = 0.006 **(Fig 4C)**. Meanwhile, a strong heterogeneity was presented in Class Ⅲ group (*I*^2^ = 93%, MD = -0.41, 95% CI [-4.34 to 3.52]; *p* = 0.84), NL/VER was demonstrated a significant difference in Class Ⅲ group after excluding heterogeneity (*I*^2^ = 41%, MD = -2.67, 95% CI [-3.78 to -1.55]; *p* < 0.00001) compared to Class Ⅰ group. It is indicated that the study reported by Hedayati *et al*. is the sources of heterogeneity and exerts an influence on the result **(Fig 4D)** [39].

#### Craniocervical posture

2 included articles measured the craniocervical angles (NSL/OPT, NSL/CVT, NL/OPT, NL/CVT) in Class Ⅰ, Ⅱ and Ⅲ malocclusion **(Fig 5)**. NL/OPT and NL/CVT showed a weak heterogeneity in Class Ⅲ group, while other variables in Class Ⅱ and Ⅲ groups exhibited a strong heterogeneity **(Fig 5A–5E and 5G)**. Although NL/CVT variables in Class Ⅲ group showed a statistical difference compared to Class I group (*I*^2^ = 0%, *p* = 0.03, MD = -1.8, 95% CI [-3.45 to -0.15]) **(Fig 5H)**, considering only 2 studies analyzed craniocervical angles (NSL/OPT, NSL/CVT, NL/OPT, NL/CVT) and a greater heterogeneity among groups, thus the analysis is not powerful enough to draw conclusions.

## Discussion

Proper posture of head and neck is maintained by a balanced tension of soft tissue (facial skin and muscles) between craniocervical bones, myofascial structures and dental occlusion according to the “soft tissue stretching” theory [34]. It is well known that muscular balance in craniofacial region plays a critical role in the development of dentofacial complex. Muscular imbalance caused by myofunctional disorders is often regarded as a contributing factor for abnormal position of teeth and jaw bones, such as in case of mouth breathing, incorrect lip seal and infantile swallowing [44–50]. In the previous epidemiologic observations, many patients with Class Ⅱ and Ⅲ malocclusion showed an altered head and neck posture. Therefore, the hypothesis that the head and cervical posture is correlated to malocclusion was proposed and widely discussed in the field of orthodontics [12, 15, 51, 52]. Based on this hypothesis, dental practitioners may prevent or treat malocclusion through correction of poor head and cervical posture. Although a number of positive outcomes were reported to prove this hypothesis, the entire orthodontic community can still not reach a consensus on this question “Does head and cervical posture correlate to malocclusion?”, most concern has focused on the design in the present studies, which may compromise the reliability of conclusion. To assist orthodontist to make accurate judgment on this issue, there is a great demand for a systematic qualitative and quantitative assessment on the relevant evidence in electronic databases.

Currently, the available data regarding the association of head and cervical posture with malocclusion was mainly obtained from the cephalometric measurement rather than experimental research. Until now, nearly thirty variables [40] were developed to determine the association of head and cervical posture with craniofacial morphology and malocclusion, of which only nine cephalometric variables representing the craniovertical, cervicohorizontal and craniocervical postural relationships and cervical column curvature (NSL/VER, NL/VER, OPT/HOR, CVT/HOR, NSL/OPT, NSL/CVT, NL/OPT, NL/CVT, OPT/CVT) were widely accepted and applied for relevant measurement. However, the craniofacial and dentofacial development is a complex and dynamic process. The current parameters obtained from 2-dimensional measurement are far from the comprehensive evaluation of the position relationship between each component in craniofacial region. With the wide application of come beam computed tomography (CBCT) and raster-stereography technique in dental clinics, more postural variables based on 3-dimensional measurement are needed to take into account, which may better reflect the association of head and cervical posture with malocclusion during development stage [21, 53]. Risk of bias is the possibility that characteristics of study design or conduct of the study will give misleading results. Generally, ethnicity, gender and age are the potential impact factors for the development of head and cervical posture [24, 25, 54, 55]. To eliminate risk of bias caused by these factors, they should be carefully examined when designing clinical studies. In the current study, 6 cross-sectional studies were finally screened out and synthesized in the meta-analysis after systematic review and identification. Unfortunately, no studies have discussed all of these influence factors. 2 studies mentioned the ethnicity when selecting subject [39, 41]. Only one study set gender subgroup and compared the difference between and in gender subgroup, although no significant differences were observed between boys and girls [38]. Moreover, most of articles did not discuss the age subgroup. Since the development of children may be affected by various genetic and environmental factors, studies without consideration of these factors may lead to bias in data interpretation. It is noteworthy that a previous study clearly pointed out that the abnormal jaw relation may be ascribed to the developmental disorder rather than poor cervical posture. Therefore, a great heterogeneity among the studies was observed in the meta-analysis, suggesting the low power of the available studies to prove the association of head and cervical posture with malocclusion.

Besides the common confounding factors like ethnicity, gender and age, various chronic diseases closely related to the change in head and cervical posture as well as malocclusion should be also taken into account when evaluating the results of studies regarding the relationship of head and cervical posture with malocclusion. For example, the obstruction of nasopharyngeal airway caused by various diseases like adenoids, nasal allergy and obstructive sleep apnea adequacy has been reported to have a close relationship with head and cervical posture [44–46, 56]. To maintain the airway patency, the head posture will be unconsciously extended in the patients with the obstruction of nasopharyngeal airway, which is indicated by an increase of the craniocervical angles. When the obstruction caused by these respiratory diseases was cured timely, the abnormal head and cervical posture can return to its normal position, or else the head and cervical posture as well as the related malocclusion may be irreversibly formed [57, 58]. Another disease that should be paid attention is temporomandibular joint disturbance syndrome (TMJDs), which have a high prevalence in children and adolescents. Patients with TMJDs often suffer from various musculoskeletal disorders involving masticatory muscles, TMJ, and surrounding structures. Although the etiology of TMJDs is still not clarified, many studies reported that TMJDs involving dysfunction of masticatory muscles may be correlated to the development of abnormal head and cervical posture [59, 60], since TMJ is connected to the cervical region via muscles and ligaments. Moreover, it is widely accepted that there is an interrelationship between malocclusion and TMJDs [61] in the field of orthodontics. Therefore, it is necessary to consider the potential impact of chronic diseases like respiratory diseases and TMJDs on the conclusion when assessing the association of head and cervical posture with malocclusion. According to the systematic review, all included studies considered the potential impact of these diseases when setting inclusion criteria, but the exclusion method was mainly based on the medical records, so the patients without obvious symptoms or signs in the latent stage of diseases may be included, probably resulting in the misinterpretation of data. With the advancement of medical technology, non-invasive techniques like Cone Beam Computed Tomography (CBCT), Magnetic Resonance Imaging (MRI), and Electromyogram (EMG) have been successfully applied for diagnose of nasopharyngeal diseases or TMJDs in their latent period. Therefore, to provide more objective evidence when grouping subjects, examination method such as CBCT, MRI and EMG are recommended in the future studies, which may be beneficial for the uniformity control of subjects.

### Limitations and strengths

Based on the above discussion, this study has two major limitations. Firstly, although all of the included studies were of moderate quality, the evidence obtained from the included studies is far from the high-quality standards for clinical trials, which was revealed in the results of this meta-analysis. As shown in **Figs 2 and 4**, large heterogeneity was observed in OPT/CVT, NSL/VER and NL/VER, which significantly affect the results of OPT/CVT in Class Ⅲ group. After excluding the source of heterogeneity in OPT/CVT, NSL/VER and NL/VER of Class Ⅲ group, the results showed a significant difference, but the valid number of participants was simultaneously reduced, which may result in the compromised reliability of data interpretation. Furthermore, no significant differences between cervical posture and Class Ⅰ, Ⅱ, and Ⅲ malocclusion were found **(Fig 3)**. Although the change of NL/VER in Class Ⅱ group showed a significant difference compared to Class Ⅰ group **(Fig 4C)**, it was not sufficient to obtain a rigorous conclusion, because only two variables of head posture were eligible. Secondly, since most of studies did not distinguish the potential confounding factors like age, gender and ethnicity among groups, no subgroup analysis was performed. Therefore, further investigations including clinical trial and experimental research are needed to prove the hypothesis regarding the relationship of head and cervical posture with malocclusion. To address the issues found in the present documents, future studies are recommended to introduce techniques like CBCT, MRI, raster-stereography or EMG to assist subject inclusion and exclusion. Moreover, researchers should be more critical in subject grouping and sample size estimation to minimize heterogeneity caused by various confounding factors.

## Conclusion

Due to the low methodological quality, race difference, and small sample sizes, the results from studies included in our review must be interpreted with caution. Findings of this systematic review and meta-analysis suggested that head and cervical posture may be correlated to Class Ⅱ and Ⅲ malocclusion, however, the current available evidence is not sound enough to support this conclusion. Therefore, the clinical practitioners should pay attention when applying any methods based on this hypothesis to treat sagittal malocclusion via correction of abnormal head and cervical posture. To better clarify the role of head and cervical posture on the development of sagittal malocclusion, large scale prospective studies with high methodological quality and minimal heterogeneity are recommended in the future.

## Supporting information

S1 Appendix Supplementary material. (DOCX)

## References

[1] Michelotti A, Manzo P, Farella M, Martina R. [Occlusion and posture: is there evidence of correlation?]. *Minerva Stomatol*. 1999;48(11):525–534. . 10768011

[2] Korbmacher H, Eggers-Stroeder G, Koch L, Kahl-Nieke B. Correlations between dentition anomalies and diseases of the of the postural and movement apparatus—a literature review. *J Orofac Orthop*. 2004;65(3):190–203. doi: 10.1007/s00056-004-0305-3 15160246

[3] Motoyoshi M, Shimazaki T, Sugai T, Namura S. Biomechanical influences of head posture on occlusion: an experimental study using finite element analysis. Eur J Orthod. 2002;24(4):319–326. doi: 10.1093/ejo/24.4.319 . 12198861

[4] Ferrillo M, Ammendolia A, Paduano S, Calafiore D, Marotta N, Migliario M, et al. Efficacy of rehabilitation on reducing pain in muscle-related temporomandibular disorders: A systematic review and meta-analysis of randomized controlled trials. *J Back Musculoskelet Rehabil*. 2022(Preprint):1–16. doi: 10.3233/BMR-210236 . 35213347

[5] Nicolakis P, Nicolakis M, Piehslinger E, Ebenbichler G, Vachuda M, Kirtley C, et al. Relationship between craniomandibular disorders and poor posture. *Cranio*. 2000;18(2):106–112. doi: 10.1080/08869634.2000.11746121 . 11202820

[6] Xu L, Zhang L, Lu J, Fan S, Cai B, Dai K. Head and neck posture influences masticatory muscle electromyographic amplitude in healthy subjects and patients with temporomandibular disorder: a preliminary study. *Ann Palliat Med*. 2021;10(3):2880–2888. doi: 10.21037/apm-20-1850 . 33691457

[7] Lieberman MA, Gazit E, Fuchs C, Lilos P. Mandibular dysfunction in 10–18 year old school children as related to morphological malocclusion. *J Oral Rehabil*. 1985;12(3):209–214. doi: 10.1111/j.1365-2842.1985.tb00637.x . 3859626

[8] Riolo ML, Brandt D, TenHave TR. Associations between occlusal characteristics and signs and symptoms of TMJ dysfunction in children and young adults. *Am J Orthod Dentofacial Orthop*. 1987;92(6):467–477. doi: 10.1016/0889-5406(87)90228-9 . 3500634

[9] Solow B, Tallgren A. Dentoalveolar morphology in relation to craniocervical posture. *Angle Orthod*. 1977;47(3):157–164. doi: 10.1043/0003-3219(1977)047&lt;0157:DMIRTC&gt;2.0.CO;2 . 268948

[10] Vukicevic V, Petrovic D. Relationship between Head Posture and Parameters of Sagittal Position and Length of Jaws. *Med Pregl*. 2016;69(9–10):288–293. doi: 10.2298/mpns1610288v . 29693851

[11] Sandoval C, Diaz A, Manriquez G. Relationship between craniocervical posture and skeletal class: A statistical multivariate approach for studying Class II and Class III malocclusions. *Cranio*. 2021;39(2):133–140. doi: 10.1080/08869634.2019.1603795 . 31035911

[12] D’Attilio M, Caputi S, Epifania E, Festa F, Tecco S. Evaluation of cervical posture of children in skeletal class I, II, and III. *Cranio*. 2005;23(3):219–228. doi: 10.1179/crn.2005.031 . 16128357

[13] Nobili A, Adversi R. Relationship between posture and occlusion: a clinical and experimental investigation. *Cranio*. 1996;14(4):274–285. doi: 10.1080/08869634.1996.11745978 . 9110621

[14] Gadotti IC, Berzin F, Biasotto-Gonzalez D. Preliminary rapport on head posture and muscle activity in subjects with class I and II. *J Oral Rehabil*. 2005;32(11):794–799. doi: 10.1111/j.1365-2842.2005.01508.x . 16202042

[15] AlKofide EA, AlNamankani E. The association between posture of the head and malocclusion in Saudi subjects. *Cranio*. 2007;25(2):98–105. doi: 10.1179/crn.2007.016 . 17508630

[16] Solow B, Sonnesen L. Head posture and malocclusions. *Eur J Orthod*. 1998;20(6):685–693. doi: 10.1093/ejo/20.6.685 . 9926635

[17] Festa F, Tecco S, Dolci M, Ciufolo F, Di Meo S, Filippi MR, et al. Relationship between cervical lordosis and facial morphology in Caucasian women with a skeletal class II malocclusion: a cross-sectional study. *Cranio*. 2003;21(2):121–129. doi: 10.1080/08869634.2003.11746240 . 12723858

[18] Ferrillo M, Marotta N, Giudice A, Calafiore D, Curci C, Fortunato L, et al. Effects of Occlusal Splints on Spinal Posture in Patients with Temporomandibular Disorders: A Systematic Review. *Healthcare (Basel)*. 2022;10(4):739. doi: 10.3390/healthcare10040739 . 35455916 PMC9027546

[19] AlGadhib F, AlQahtani R, AlBej R, AlOtaibi W, AlFakih E, Ateeq IS. Design of a Smart Posture Corrective Orthosis for Kyphotic Patients. *CMBES Proceedings*. 2021;44. Available from: https://proceedings.cmbes.ca/index.php/proceedings/article/view/947

[20] Kayiran T, Turhan B. The effectiveness of neural mobilization in addition to conservative physiotherapy on cervical posture, pain and functionality in patients with cervical disc herniation. *Advances in Rehabilitation*. 2021;35(3):8–16. 10.5114/areh.2021.107788

[21] Lippold C, Danesh G, Schilgen M, Drerup B, Hackenberg L. Sagittal jaw position in relation to body posture in adult humans—a rasterstereographic study. *BMC Musculoskelet Disord*. 2006;7(1):8. doi: 10.1186/1471-2474-7-8 . 16448563 PMC1379641

[22] Perinetti G, Contardo L, Silvestrini-Biavati A, Perdoni L, Castaldo A. Dental malocclusion and body posture in young subjects: a multiple regression study. *Clinics (Sao Paulo)*. 2010;65(7):689–695. doi: 10.1590/S1807-59322010000700007 . 20668626 PMC2910857

[23] Gomes Lde C, Horta KO, Goncalves JR, Santos-Pinto AD. Systematic review: craniocervical posture and craniofacial morphology. *Eur J Orthod*. 2014;36(1):55–66. doi: 10.1093/ejo/cjt004 . 23612566

[24] Hellsing E, Reigo T, McWilliam J, Spangfort E. Cervical and lumbar lordosis and thoracic kyphosis in 8, 11 and 15-year-old children. *Eur J Orthod*. 1987;9(2):129–138. doi: 10.1093/ejo/9.2.129 . 3472891

[25] Tecco S, Festa F. Cervical spine curvature and craniofacial morphology in an adult Caucasian group: a multiple regression analysis. *Eur J Orthod*. 2007;29(2):204–209. doi: 10.1093/ejo/cjl061 . 17218718

[26] Pacella E, Dari M, Giovannoni D, Mezio M, Caterini L, Costantini AM, et al. The relationship between occlusion and posture: A systematic review. 2017. WebmedCentral ORTHODONTICS:[WMC005374] Available from: http://www.webmedcentral.com/article_view/537

[27] Huggare J. Postural disorders and dentofacial morphology. *Acta Odontol Scand*. 1998;56(6):383–386. doi: 10.1080/000163598428374 . 10066122

[28] Michelotti A, Buonocore G, Manzo P, Pellegrino G, Farella M. Dental occlusion and posture: an overview. *Prog Orthod*. 2011;12(1):53–58. doi: 10.1016/j.pio.2010.09.010 . 21515232

[29] Rodrigues CDA, Neto HP, Silva ACdO, Stancker TG. Malocclusion influence on balance and posture: a systematic review. *MTP&RehabJournal*. 2015:1–6. 10.17784/mtprehabJournal.2015.13.320

[30] Cooke MS. Five-year reproducibility of natural head posture: a longitudinal study. *Am J Orthod Dentofacial Orthop*. 1990;97(6):489–494. doi: 10.1016/S0889-5406(05)80029-0 . 2353678

[31] Jakobsone G, Vuollo V, Pirttiniemi P. Reproducibility of Natural Head Position assessed with stereophotogrammetry. *Orthod Craniofac Res*. 2020;23(1):66–71. doi: 10.1111/ocr.12344 . 31514260

[32] Peng L, Cooke MS. Fifteen-year reproducibility of natural head posture: A longitudinal study. *Am J Orthod Dentofacial Orthop*. 1999;116(1):82–85. doi: 10.1016/s0889-5406(99)70306-9 . 10393584

[33] Weber DW, Fallis DW, Packer MD. Three-dimensional reproducibility of natural head position. *Am J Orthod Dentofacial Orthop*. 2013;143(5):738–744. doi: 10.1016/j.ajodo.2012.11.026 . 23631976

[34] Solow B, Tallgren A. Head posture and craniofacial morphology. *Am J Phys Anthropol*. 1976;44(3):417–435. doi: 10.1002/ajpa.1330440306 . 937521

[35] Pearson A, Jordan Z. Evidence-based healthcare in developing countries. *Int J Evid Based Healthc*. 2010;8(2):97–100. 10.1111/j.1744-1609.2010.00164.x . 21077397

[36] Higgins JP, Thompson SG. Quantifying heterogeneity in a meta-analysis. *Stat Med*. 2002;21(11):1539–1558. doi: 10.1002/sim.1186 . 12111919

[37] Higgins JP, Thompson SG, Deeks JJ, Altman DG. Measuring inconsistency in meta-analyses. *BMJ*. 2003;327(7414):557–560. doi: 10.1136/bmj.327.7414.557 . 12958120 PMC192859

[38] Bernal LV, Marin H, Herrera CP, Montoya C, Herrera YU. Craniocervical posture in children with class I, II and III skeletal relationships. *Pesq Bras Odontoped Clin Integr*. 2017;17(1):1–12. 10.4034/PBOCI.2017.171.07

[39] Hedayati Z, Paknahad M, Zorriasatine F. Comparison of natural head position in different anteroposterior malocclusions. *J Dent (Tehran)*. 2013;10(3):210–220. . 25512747 PMC4264092

[40] Liu Y, Sun X, Chen Y, Hu M, Hou X, Liu C. Relationships of sagittal skeletal discrepancy, natural head position, and craniocervical posture in young Chinese children. *Cranio*. 2016;34(3):155–162. doi: 10.1179/2151090315Y.0000000015 . 26039882

[41] Hosseinzadeh Nik T, Janbaz Aciyabar P. The relationship between cervical column curvature and sagittal position of the jaws: using a new method for evaluating curvature. *Iran J Radiol*. 2011;8(3):161–166. doi: 10.5812/kmp.iranjradiol.17351065.3379 . 23329934 PMC3522334

[42] Qadir M, Mushtaq M. Cephalometric evaluation of cervical column curvature with respect to sagittal jaw position. *Am J Phys Anthropol*. 2017;3:238–242.

[43] Tauheed S, Shaikh A, Fida M. Cervical posture and skeletal malocclusions–is there a link? *JCMS Nepal*. 2019;15(1):5–9. 10.3126/JCMSN.V15I1.20509

[44] Ozbek MM, Miyamoto K, Lowe AA, Fleetham JA. Natural head posture, upper airway morphology and obstructive sleep apnoea severity in adults. *Eur J Orthod*. 1998;20(2):133–143. doi: 10.1093/ejo/20.2.133 . 9633167

[45] Chambi-Rocha A, Cabrera-Dominguez ME, Dominguez-Reyes A. Breathing mode influence on craniofacial development and head posture. *J Pediatr (Rio J)*. 2018;94(2):123–130. doi: 10.1016/j.jped.2017.05.007 . 28818510

[46] Chaves TC, de Andrade e Silva TS, Monteiro SA, Watanabe PC, Oliveira AS, Grossi DB. Craniocervical posture and hyoid bone position in children with mild and moderate asthma and mouth breathing. *Int J Pediatr Otorhinolaryngol*. 2010;74(9):1021–1027. doi: 10.1016/j.ijporl.2010.05.031 . 20566222

[47] Priede D, Roze B, Parshutin S, Arklina D, Pircher J, Vaska I, et al. Association between malocclusion and orofacial myofunctional disorders of pre-school children in Latvia. *Orthod Craniofac Res*. 2020;23(3):277–283. doi: 10.1111/ocr.12367 . 31989782

[48] Grabowski R, Stahl F, Gaebel M, Kundt G. [Relationship between occlusal findings and orofacial myofunctional status in primary and mixed dentition. Part I: Prevalence of malocclusions]. *J Orofac Orthop*. 2007;68(1):26–37. doi: 10.1007/s00056-007-1606-0 . 17238051

[49] Nanda SB, Sinha A, Mali L, Acharya SS. Effect of Naso-respiratory Obstruction with Mouth Breathing on Dentofacial and Craniofacial Development. *Orthodontic Journal of Nepal*. 2018;8(1):22–27. 10.3126/ojn.v8i1.21343

[50] Conti PB, Sakano E, Ribeiro MA, Schivinski CI, Ribeiro JD. Assessment of the body posture of mouth-breathing children and adolescents. *J Pediatr (Rio J)*. 2011;87(4):357–363. doi: 10.2223/JPED.2102 . 21769416

[51] Arntsen T, Sonnesen L. Cervical vertebral column morphology related to craniofacial morphology and head posture in preorthodontic children with Class II malocclusion and horizontal maxillary overjet. *Am J Orthod Dentofacial Orthop*. 2011;140(1):e1–7. doi: 10.1016/j.ajodo.2010.10.021 . 21724066

[52] Gonzalez HE, Manns A. Forward head posture: its structural and functional influence on the stomatognathic system, a conceptual study. *Cranio*. 1996;14(1):71–80. doi: 10.1080/08869634.1996.11745952 . 9086879

[53] Watanabe M, Yamaguchi T, Maki K. Cervical vertebra morphology in different skeletal classes. A three-dimensional computed tomography evaluation. *Angle Orthod*. 2010;80(4):531–536. doi: 10.2319/100609-557.1 . 20482359 PMC8966444

[54] Grave B, Brown T, Townsend G. Comparison of cervicovertebral dimensions in Australian Aborigines and Caucasians. *Eur J Orthod*. 1999;21(2):127–135. doi: 10.1093/ejo/21.2.127 . 10327736

[55] García N, Sanhueza A, Cantín M, Fuentes R. Evaluación de la Postura Cervical en Sujetos Adolescentes con Clase Esqueletal I, II y III. *Int j morphol*. 2012;30(2):405–410. 10.4067/S0717-95022012000200007

[56] Solow B, Ovesen J, Nielsen PW, Wildschiodtz G, Tallgren A. Head posture in obstructive sleep apnoea. *Eur J Orthod*. 1993;15(2):107–114. doi: 10.1093/ejo/15.2.107 . 8500536

[57] Makofsky HW. Snoring and obstructive sleep apnea: does head posture play a role? *Cranio*. 1997;15(1):68–73. doi: 10.1080/08869634.1997.11745994 . 9586490

[58] Tong M, Sakakibara H, Xia X, Suetsugu S. Compensatory head posture changes in patients with obstructive sleep apnea. *J Tongji Med Univ*. 2000;20(1):66–69. doi: 10.1007/BF02887681 . 12845762

[59] Evcik D, Aksoy O. Relationship between head posture and temporomandibular dysfunction syndrome. *MYOPAIN*. 2004;12(2):19–24. 10.1300/J094v12n02_03

[60] Sonnesen L, Bakke M, Solow B. Temporomandibular disorders in relation to craniofacial dimensions, head posture and bite force in children selected for orthodontic treatment. *Eur J Orthod*. 2001;23(2):179–192. doi: 10.1093/ejo/23.2.179 . 11398555

[61] Runge ME, Sadowsky C, Sakols EI, BeGole EA. The relationship between temporomandibular joint sounds and malocclusion. *Am J Orthod Dentofacial Orthop*. 1989;96(1):36–42. doi: 10.1016/0889-5406(89)90226-6 . 2750718

