# Supplementary material for: Does head and cervical posture correlate to malocclusion? A systematic review and meta-analysis
Source: PLoS One. 2022 Oct 25;17(10):e0276156. doi: 10.1371/journal.pone.0276156 (PMC9595508; doi:10.1371/journal.pone.0276156)
Supplement: S1 Appendix — (DOCX) [file pone.0276156.s001.docx]

**Supplementary material Appendix 1**

**Table of Contents:**

**Table A.** PRISMA checklist

**Table B.** Search strategy

**Table C.** Reference Points, Lines and Angles on the Cephalograms

**Fig A.** Cephalometric tracings and variables studied in our study

**Table A.** PRISMA checklist

| **Section and Topic** | **Item #** | **Checklist item** | **Location where item is reported** |
| --- | --- | --- | --- |
| **TITLE** | | |  |
| Title | 1 | Identify the report as a systematic review. | p.1 |
| **ABSTRACT** | | |  |
| Abstract | 2 | See the PRISMA 2020 for Abstracts checklist. | p.2-3 |
| **INTRODUCTION** | | |  |
| Rationale | 3 | Describe the rationale for the review in the context of existing knowledge. | p.4-5 |
| Objectives | 4 | Provide an explicit statement of the objective(s) or question(s) the review addresses. | p.5-6 |
| **METHODS** | | |  |
| Eligibility criteria | 5 | Specify the inclusion and exclusion criteria for the review and how studies were grouped for the syntheses. | p.7-8 |
| Information sources | 6 | Specify all databases, registers, websites, organisations, reference lists and other sources searched or consulted to identify studies. Specify the date when each source was last searched or consulted. | p.6-7 |
| Search strategy | 7 | Present the full search strategies for all databases, registers and websites, including any filters and limits used. | p.6-7 |
| Selection process | 8 | Specify the methods used to decide whether a study met the inclusion criteria of the review, including how many reviewers screened each record and each report retrieved, whether they worked independently, and if applicable, details of automation tools used in the process. | p.8-9 |
| Data collection process | 9 | Specify the methods used to collect data from reports, including how many reviewers collected data from each report, whether they worked independently, any processes for obtaining or confirming data from study investigators, and if applicable, details of automation tools used in the process. | p.8-9 |
| Data items | 10a | List and define all outcomes for which data were sought. Specify whether all results that were compatible with each outcome domain in each study were sought (e.g. for all measures, time points, analyses), and if not, the methods used to decide which results to collect. | p.9 |
|  | 10b | List and define all other variables for which data were sought (e.g. participant and intervention characteristics, funding sources). Describe any assumptions made about any missing or unclear information. | p.9 |
| Study risk of bias assessment | 11 | Specify the methods used to assess risk of bias in the included studies, including details of the tool(s) used, how many reviewers assessed each study and whether they worked independently, and if applicable, details of automation tools used in the process. | p. 9-10 |
| Effect measures | 12 | Specify for each outcome the effect measure(s) (e.g. risk ratio, mean difference) used in the synthesis or presentation of results. | p.10-11 |
| Synthesis methods | 13a | Describe the processes used to decide which studies were eligible for each synthesis (e.g. tabulating the study intervention characteristics and comparing against the planned groups for each synthesis (item #5)). | p.9 |
|  | 13b | Describe any methods required to prepare the data for presentation or synthesis, such as handling of missing summary statistics, or data conversions. | p.10-11 |
|  | 13c | Describe any methods used to tabulate or visually display results of individual studies and syntheses. | p.10-11 |
|  | 13d | Describe any methods used to synthesize results and provide a rationale for the choice(s). If meta-analysis was performed, describe the model(s), method(s) to identify the presence and extent of statistical heterogeneity, and software package(s) used. | p.10-11 |
|  | 13e | Describe any methods used to explore possible causes of heterogeneity among study results (e.g. subgroup analysis, meta-regression). | p.10-11 |
|  | 13f | Describe any sensitivity analyses conducted to assess robustness of the synthesized results. | - |
| Reporting bias assessment | 14 | Describe any methods used to assess risk of bias due to missing results in a synthesis (arising from reporting biases). | p.11 |
| Certainty assessment | 15 | Describe any methods used to assess certainty (or confidence) in the body of evidence for an outcome. | - |
| **RESULTS** | | |  |
| Study selection | 16a | Describe the results of the search and selection process, from the number of records identified in the search to the number of studies included in the review, ideally using a flow diagram. | p.11-12 |
|  | 16b | Cite studies that might appear to meet the inclusion criteria, but which were excluded, and explain why they were excluded. | p.12 |
| Study characteristics | 17 | Cite each included study and present its characteristics. | p.12,14 |
| Risk of bias in studies | 18 | Present assessments of risk of bias for each included study. | p.13,15 |
| Results of individual studies | 19 | For all outcomes, present, for each study: (a) summary statistics for each group (where appropriate) and (b) an effect estimate and its precision (e.g. confidence/credible interval), ideally using structured tables or plots. | p.16 |
| Results of syntheses | 20a | For each synthesis, briefly summarise the characteristics and risk of bias among contributing studies. | p.16-19 |
|  | 20b | Present results of all statistical syntheses conducted. If meta-analysis was done, present for each the summary estimate and its precision (e.g. confidence/credible interval) and measures of statistical heterogeneity. If comparing groups, describe the direction of the effect. | p.16-19 |
|  | 20c | Present results of all investigations of possible causes of heterogeneity among study results. | p.16-19 |
|  | 20d | Present results of all sensitivity analyses conducted to assess the robustness of the synthesized results. | p.16-19 |
| Reporting biases | 21 | Present assessments of risk of bias due to missing results (arising from reporting biases) for each synthesis assessed. | p.16 |
| Certainty of evidence | 22 | Present assessments of certainty (or confidence) in the body of evidence for each outcome assessed. | - |
| **DISCUSSION** | | |  |
| Discussion | 23a | Provide a general interpretation of the results in the context of other evidence. | p.19-23 |
|  | 23b | Discuss any limitations of the evidence included in the review. | p.23-24 |
|  | 23c | Discuss any limitations of the review processes used. | p.23-24 |
|  | 23d | Discuss implications of the results for practice, policy, and future research. | p.23-24 |
| **OTHER INFORMATION** | | |  |
| Registration and protocol | 24a | Provide registration information for the review, including register name and registration number, or state that the review was not registered. | p.6 |
|  | 24b | Indicate where the review protocol can be accessed, or state that a protocol was not prepared. | p.6 |
|  | 24c | Describe and explain any amendments to information provided at registration or in the protocol. | - |
| Support | 25 | Describe sources of financial or non-financial support for the review, and the role of the funders or sponsors in the review. | p.24-25 |
| Competing interests | 26 | Declare any competing interests of review authors. | p.1 |
| Availability of data, code and other materials | 27 | Report which of the following are publicly available and where they can be found: template data collection forms; data extracted from included studies; data used for all analyses; analytic code; any other materials used in the review. | p.25 |

*From:*  Page MJ, McKenzie JE, Bossuyt PM, Boutron I, Hoffmann TC, Mulrow CD, et al. The PRISMA 2020 statement: an updated guideline for reporting systematic reviews. BMJ 2021;372:n71. doi: 10.1136/bmj.n71. For more information, visit: http://www.prisma-statement.org/

**Table B.** Search strategy

| **Datebase** | **Search term** | **Result** |
| --- | --- | --- |
| Pubmed | ("malocclusal"[All Fields] OR "malocclusion"[MeSH Terms] OR "malocclusion"[All Fields] OR "malocclusions"[All Fields] OR "malocclusive"[All Fields] OR ("crowd s"[All Fields] OR "crowding"[MeSH Terms] OR "crowding"[All Fields] OR "crowd"[All Fields] OR "crowded"[All Fields] OR "crowds"[All Fields]) OR ("malocclusion"[MeSH Terms] OR "malocclusion"[All Fields] OR "crossbite"[All Fields] OR "crossbites"[All Fields]) OR ("malocclusion"[MeSH Terms] OR "malocclusion"[All Fields] OR ("angles"[All Fields] AND "classification"[All Fields]) OR "angles classification"[All Fields]) OR ("overbite"[MeSH Terms] OR "overbite"[All Fields] OR "overbites"[All Fields]) OR ("open bite"[MeSH Terms] OR ("open"[All Fields] AND "bite"[All Fields]) OR "open bite"[All Fields]) OR (("angle"[All Fields] OR "angled"[All Fields] OR "angles"[All Fields]) AND ("malocclusion, angle class ii"[MeSH Terms] OR ("malocclusion"[All Fields] AND "angle"[All Fields] AND "class"[All Fields] AND "ii"[All Fields]) OR "angle class ii malocclusion"[All Fields] OR ("class"[All Fields] AND "ii"[All Fields] AND "malocclusion"[All Fields]) OR "class ii malocclusion"[All Fields])) OR (("malocclusion, angle class iii"[MeSH Terms] OR ("malocclusion"[All Fields] AND "angle"[All Fields] AND "class"[All Fields] AND "iii"[All Fields]) OR "angle class iii malocclusion"[All Fields] OR ("angle"[All Fields] AND "class"[All Fields] AND "iii"[All Fields]) OR "angle class iii"[All Fields]) AND ("malocclusal"[All Fields] OR "malocclusion"[MeSH Terms] OR "malocclusion"[All Fields] OR "malocclusions"[All Fields] OR "malocclusive"[All Fields]))) AND ((("head"[MeSH Terms] OR "head"[All Fields]) AND ("postural"[All Fields] OR "posturally"[All Fields] OR "posture"[MeSH Terms] OR "posture"[All Fields] OR "postures"[All Fields] OR "postured"[All Fields] OR "posturing"[All Fields])) OR (("cervic"[All Fields] OR "cervicals"[All Fields] OR "cervices"[All Fields] OR "neck"[MeSH Terms] OR "neck"[All Fields] OR "cervical"[All Fields] OR "uterine cervicitis"[MeSH Terms] OR ("uterine"[All Fields] AND "cervicitis"[All Fields]) OR "uterine cervicitis"[All Fields] OR "cervicitis"[All Fields]) AND ("postural"[All Fields] OR "posturally"[All Fields] OR "posture"[MeSH Terms] OR "posture"[All Fields] OR "postures"[All Fields] OR "postured"[All Fields] OR "posturing"[All Fields])) OR (("cervical vertebrae"[MeSH Terms] OR ("cervical"[All Fields] AND "vertebrae"[All Fields]) OR "cervical vertebrae"[All Fields]) AND ("postural"[All Fields] OR "posturally"[All Fields] OR "posture"[MeSH Terms] OR "posture"[All Fields] OR "postures"[All Fields] OR "postured"[All Fields] OR "posturing"[All Fields])) OR (("cervical vertebrae"[MeSH Terms] OR ("cervical"[All Fields] AND "vertebrae"[All Fields]) OR "cervical vertebrae"[All Fields] OR ("cervical"[All Fields] AND "vertebra"[All Fields]) OR "cervical vertebra"[All Fields]) AND ("postural"[All Fields] OR "posturally"[All Fields] OR "posture"[MeSH Terms] OR "posture"[All Fields] OR "postures"[All Fields] OR "postured"[All Fields] OR "posturing"[All Fields])) OR (("neck"[MeSH Terms] OR "neck"[All Fields]) AND ("postural"[All Fields] OR "posturally"[All Fields] OR "posture"[MeSH Terms] OR "posture"[All Fields] OR "postures"[All Fields] OR "postured"[All Fields] OR "posturing"[All Fields])) OR (("head"[MeSH Terms] OR "head"[All Fields]) AND ("patient positioning"[MeSH Terms] OR ("patient"[All Fields] AND "positioning"[All Fields]) OR "patient positioning"[All Fields] OR "positioning"[All Fields] OR "position"[All Fields] OR "position s"[All Fields] OR "positional"[All Fields] OR "positioned"[All Fields] OR "positionings"[All Fields] OR "positions"[All Fields]))) | 1071 |
| Embase | malocclusions OR 'malocclusion'/exp OR malocclusion OR 'crowding'/exp OR crowding OR 'crossbite'/exp OR crossbite OR 'angle class ii malocclusion'/exp OR 'angle class ii malocclusion' OR (angle AND class AND ii AND ('malocclusion'/exp OR malocclusion)) OR 'angle class iii malocclusion'/exp OR 'angle class iii malocclusion' OR (angle AND class AND iii AND ('malocclusion'/exp OR malocclusion)) OR 'jaw occlusion disorder'/exp OR 'jaw occlusion disorder' OR (('jaw'/exp OR jaw) AND ('occlusion'/exp OR occlusion) AND ('disorder'/exp OR disorder)) OR 'open bite'/exp OR 'open bite' OR (open AND ('bite'/exp OR bite)) OR 'overbite'/exp OR overbite AND 'head posture'/exp OR 'head posture' OR (('head'/exp OR head) AND ('posture'/exp OR posture)) OR 'neck posture'/exp OR 'neck posture' OR (('neck'/exp OR neck) AND ('posture'/exp OR posture)) OR 'cervical vertebrae posture' OR (cervical AND vertebrae AND ('posture'/exp OR posture)) OR 'cervical vertebra posture' OR (cervical AND ('vertebra'/exp OR vertebra) AND ('posture'/exp OR posture)) OR 'cervical posture' OR (cervical AND ('posture'/exp OR posture)) | 436 |
| Cochrane | (cervical vertebra posture):ti,ab,kw OR (cervical vertebrae posture) OR (cervical posture) OR (head posture)OR (neck posture) (Word variations have been searched)AND(malocclusion):ti,ab,kw OR (crowding) OR (overbite):kw OR (crossbite) OR (open bite) (Word variations have been searched) | 39 |

**Table C.** Reference Points, Lines and Angles on the Cephalograms

|  | **Description** | **Characterization** |
| --- | --- | --- |
| **Reference point** |  |  |
| S | Sella turcica | The center of the Sella turcica (1) |
| N | Nasion | The most anterior point of frontonasal suture (1) |
| O | Orbitale | The lowest point on the lower margin of the bony orbit (2) |
| P | Porion, | The most superior point of bony external auditory meatus (2) |
| ANS | Anterior Nasal Spine | The most anterior point of nasal spine (2) |
| PNS | Posterio Nasal Spine | The most posterior point of nasal spine (2) |
| CV2ip |  | The most infero-posterior (ip) point of the second cervical vertebra (CV2) (1, 2) |
| CV2tg |  | The tangent (tg) point at the most superior posterior point of the second cervical vertebra (CV2) (1, 2) |
| CV4ip |  | The most infero-posterior (ip) point of the fourth cervical vertebra (CV4) (1, 2) |
| **Reference lines** |  |  |
| NSL | Nasion-Sella Line | The line through N and S points (1-3) |
| NL | Nasal Line | The line through ANS and PNS points (1, 3) |
| FH | Frankfort Horizontal | Frankfort Horizontal plane, The line through O and P points (2) |
| HOR | Horizontal | True horizontal line projected on the film (1) |
| VER | Vertical | True vertical line projected on the film (1) |
| OPT | Odontoid Process Tangent | Through Cv2tg and Cv2ip points (1, 4) |
| CVT | Cervical Vertebra Tangent | Through Cv2tg and Cv4ip points (1, 4) |
| **Reference angles** |  |  |
| NSL/OPT |  | Angle formed between the lines NSL and OPT (3) |
| NSL/CVT |  | Angle formed between the lines NSL and CVT (3) |
| NL/OPT |  | Angle formed between the lines NL and OPT (3) |
| NL/CVT |  | Angle formed between the lines NL and CVT (3) |
| OPT/CVT |  | Angle formed between the lines OPT and CVT (3) |
| CVT/HOR |  | Angle formed between the lines CVT and HOR (3) |
| OPT/HOR |  | Angle formed between the lines OPT and HOR (3) |
| NSL/VER |  | Angle formed between the lines NSL and VER (3) |
| NL/VER |  | Angle formed between the lines NL and VER (3) |

| 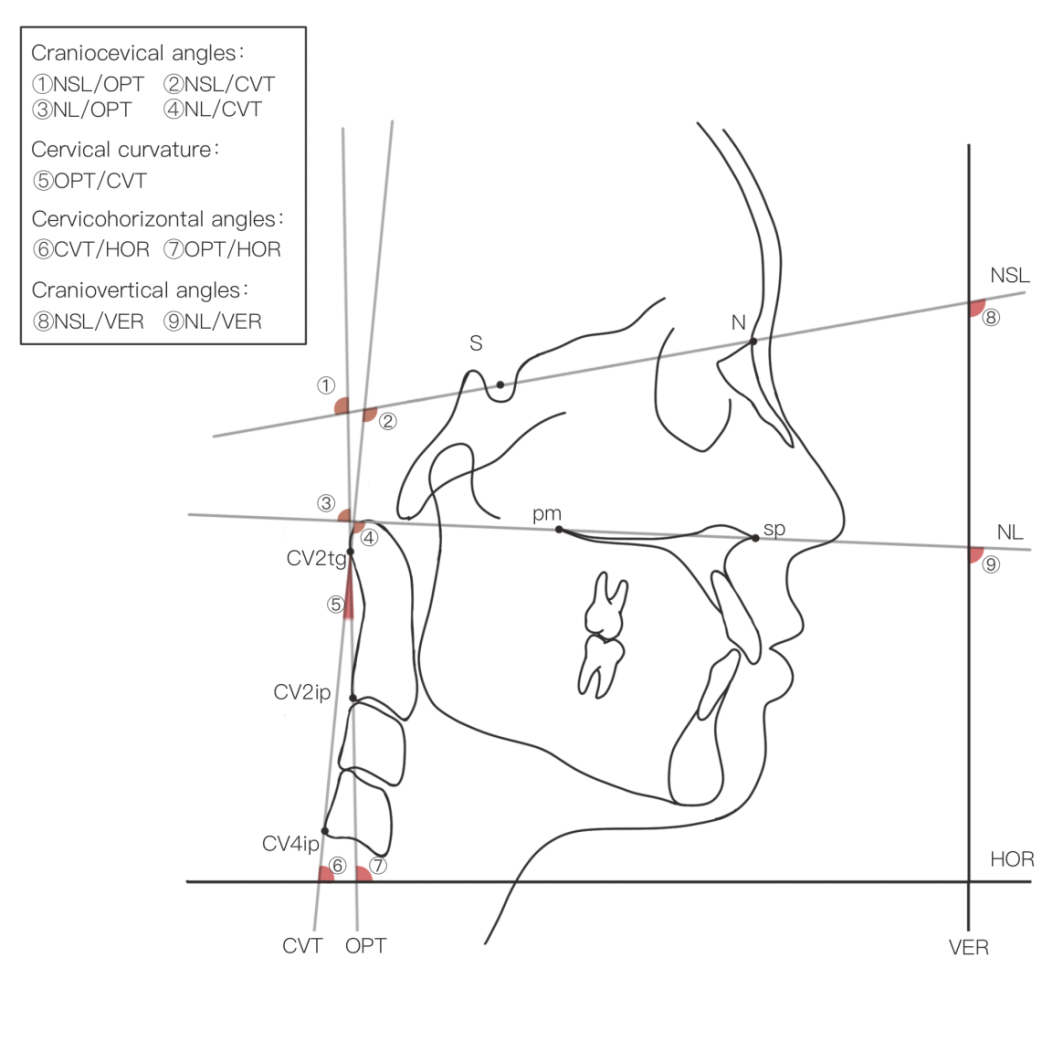 |
| --- |
| **Fig A.** Cephalometric tracings and variables studied in our study |

**References**

1. Solow B, Tallgren A. Head posture and craniofacial morphology. *Am J Phys Anthropol*. 1976;44(3):417-435.

2. D’Attilio M, Caputi S, Epifania E, Festa F, Tecco S. Evaluation of cervical posture of children in skeletal class I, II, and III. *Cranio*. 2005;23(3):219-228.

3. Liu Y, Sun X, Chen Y, Hu M, Hou X, Liu C. Relationships of sagittal skeletal discrepancy, natural head position, and craniocervical posture in young Chinese children. *Cranio*. 2016;34(3):155-162.

4. Qadir M, Mushtaq M. Cephalometric evaluation of cervical column curvature with respect to sagittal jaw position. *Am J Phys Anthropol*. 2017;3:238-242.
